# Multifunctional Gel Films of Marine Polysaccharides Cross-Linked with Poly-Metal Ions for Wound Healing

**DOI:** 10.3390/ph15060750

**Published:** 2022-06-15

**Authors:** Di Zhao, Chao Shi, Tingting Guo, Kun Zhang, Shenghao Cui, Liqi Chen, Faming Yang, Jingdi Chen

**Affiliations:** Marine College, Shandong University, Weihai 264209, China; mjqyhhdzdys@163.com (D.Z.); shichao19834031211@163.com (C.S.); gtt1314xf@163.com (T.G.); sdtzzk2021@163.com (K.Z.); csh1473@163.com (S.C.); clychee0307@163.com (L.C.)

**Keywords:** marine polysaccharide, polymetallic ion-COS chelator, casting and in-situ spray method, antibacterial properties, wound healing

## Abstract

The development of an efficient and convenient material to improve skin tissue regeneration is a major challenge in healthcare. Inspired by the theory of moist wound healing, portable chitooligosaccharide (COS)/sodium alginate (SA) dual-net gel films containing multiple metal ions were prepared by a casting and in-situ spray method, which can be used to significantly promote wound healing without the use of therapeutic drugs. A variety of divalent cations was introduced in this experiment to improve the advantages of each metal ion by forming metal ion chelates with COS. Moreover, the physicochemical properties and antioxidant properties of nIon^2+^-COS/SA gel films were systematically characterized and evaluated by in vitro experiments. The gel films showed good antibacterial activity against Gram-negative and Gram-positive bacteria. In addition, the gel films showed good cytocompatibility in cellular experiments, and the gel films with Zn^2+^ and Sr^2+^ addition significantly accelerated wound healing in whole skin defect model experiments. Therefore, this nIon^2+^-COS/SA gel film is an ideal candidate material for wound dressing.

## 1. Introduction

The skin is the largest organ of the human body, and millions of patients worldwide suffer from skin trauma every year [1]. Therefore, the study of wound healing is becoming more and more popular and important [2]. Wound healing goes through four successive phases: hemostasis, inflammation, proliferation, and remodeling. If wounds are not treated promptly and effectively, they are likely to lead to the development of chronic wounds with consequences including poor circulation, hypoxia, bacterial infections at the wound site, as well as many other problems [3]. Many treatments for wounds have been developed, including inhibiting inflammation, promoting epithelial regeneration, reducing oxidative stress, increasing oxygen levels, promoting angiogenesis, enhancing fibroblast migration, improving the bioavailability of bioactive factors and growth factors, and eliminating microbial infections in the wound [4]. However, existing treatment strategies do not fully address the functional needs of all stages of wound healing and often address only one or a few of these problems. In a nutshell, there is a need to develop multifunctional, safe, efficient, and low-cost wound dressings.

Currently, scholars at home and abroad are exploring new wound dressings, and hydrogel is the best candidate for wound dressing because of its moisturizing and drug-releasing properties [5]. COS is an oligomer of chitosan (CS), which has superior biological activity compared to CS, as well as more outstanding physicochemical properties [6]. For example, the solubility of COS is much higher than that of CS, which is easily soluble in water and does not need to be dissolved in acetic acid solution, so it has bright prospects for application in biomedical fields such as wound dressings [7]. SA, a natural anionic polysaccharide derived from brown algae, not only has a rich source, but is also non-immunogenic, with good biocompatibility and biodegradability. It exhibits simple gelation by interacting with divalent cations and related studies have been reported [8]. Unfortunately, the hydrogels prepared in this way usually do not have adequate mechanical properties [9]. Although double -network hydrogels can effectively solve this problem, the use of chemical cross-linking agents usually destroys the biocompatibility of hydrogels and affects their biodegradability [10]. In other words, it is particularly important to develop fully physically cross-linked natural double net hydrogels [11].

Here, we prepared versatile nIon^2+^-COS/SA physically crosslinked double-network hydrogel film (abbreviated as COS/SA/nM^2+^ PDF) by the casting and in -situ spray method [12]. The physicochemical properties, antimicrobial properties, antioxidant properties, as well as cytocompatibility and ability to promote wound healing of COS/SA/nM^2+^ PDF were also systematically characterized and evaluated in a series of experiments [13].

## 2. Results and Discussion

### 2.1. Preparation Mechanism of COS/SA/nM^2+^ PDF

A new method for the preparation of physically cross-linked double -network gel films was innovatively proposed in a convenient and effective manner. COS is an oligomer of CS, which has more prominent biological activity. Moreover, the water solubility of COS is very good, which greatly expands its application in many fields [14]. Here, we configured COS and complex metal ion salt into a mixed solution and adjusted the pH. Then we sprayed it evenly on the mold with an extended flow of SA solution with a spray bottle, and the cation-NH_3_^+^ of COS interacted with the anion-COO^−^ of SA electrostatically to form a COS/SA network [15]. At the same time, the complexed metal ions were chelated with the gulosonic acid blocks of alginate chains to form a SA/complexed metal ions network, which formed nIon^2+^-COS/SA physically cross-linked double network gel films in situ after a period of time [16]. This preparation method avoids the use of toxic chemical reagents and allows the preparation of COS/SA/nM^2+^ PDF with a controlled size and thickness.

### 2.2. Chemical Structure and Property Analysis of COS/SA/nM^2+^ PDF

#### 2.2.1. Surface Appearance

The pore sizes of nIon^2+^-COS/SA gel films were all small, and all films showed interconnected, uniform, fine pore structures with a certain degree of roughness [17]. Such a structure on the one hand gives COS/SA/nM^2+^ PDF a higher specific surface area and water content. On the other hand, as a wound dressing it is conducive to absorbing wound exudate, transporting nutrients and excreting metabolic waste, as well as providing a certain three-dimensional space for cell adhesion and proliferation [18]. 

**Figure 1:**
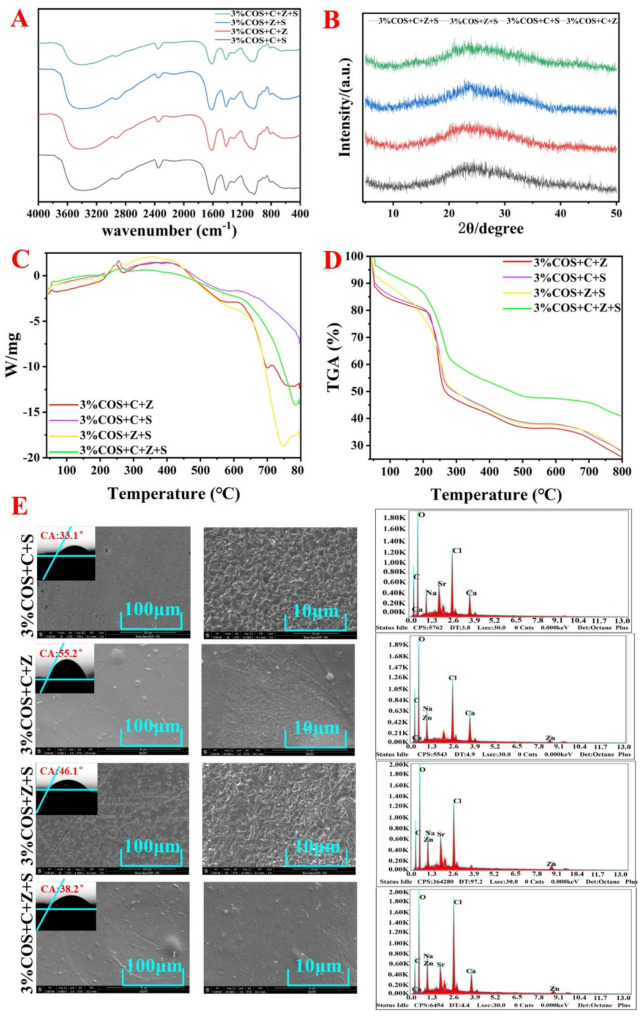
Characterization of nIon^2+^−COS/SA gel films. (**A**) FTIR spectra of nIon^2+^−COS/SA gel films. (**B**) XRD patterns of nIon^2+^−COS/SA gel films. (**C**,**D**) TGA patterns of nIon^2+^−COS/SA gel films. (**E**) SEM morphology and energy spectrum analysis of nIon^2+^−COS/SA gel films.

The SEM observation showed that the 3%COS+C+Z+S group had the densest gel structure with good dispersion, slight agglomeration, and obvious granularity; the 3%COS+C+S group had the loosest structure with poor dispersion (Figure 1E). From the valence bonding theory, it may be due to the fact that Zn^2+^ can only cross-link with the G and M sugar residues in the SA molecular chain through the orbital configuration of sp^3^ hybridization and the number of cross-linking sites is large, which results in the formation of a dense gel skeleton with a small particle size. In contrast, the planar square dsp^2^ hybridization orbital configuration of Ca^2+^ restricts it to cross-link with the GG block in SA and the number of cross-linking sites is small, which results in a loose gel skeleton with low shrinkage stress. The systolic stress is low, resulting in a larger particle size [19]. It was demonstrated that the differences in surface morphology could be attributed to the affinity of alginate chains for different divalent metal ions, resulting in differences in the sparsity of the polymer network structure.

#### 2.2.2. FTIR Analysis

Figure 1A showed that the peaks near 3400 cm^−1^ were attributed to the stretching vibrations of the O-H and N-H bonds. The peak near 2300 cm^−1^ was attributed to the symmetric stretching vibrations of -CH, and the peak at 1400 cm^−1^ reflected the symmetric deformation of -CH_3_ [20]. In addition, the absorption peaks around 1000 cm^−1^ were from the bending vibration of C=O, and the absorption peaks near 1650 cm^−1^ were attributed to the amide band. A total of 3% of the absorption peaks in the 3%COS+C+Z+S group were weak, while the 3%COS+Z+S, 3%COS+C+S, and 3%COS+C+Z groups were enhanced sequentially due to the bending vibration of the double bond over the single bond −COO and C=O in the amide group and N-H in the amide group to different degrees. When two different metal ions form a binary cross-linker, the gel film may form a unique structure different from the single ion cross-linker, due to the coexistence of the two cross-linking mechanisms and the different degrees of stretching vibrations generated by the absorption summit [21]. The above showed that −COO^−^ and −NH_3_^+^ interacted with metal ions, confirming the generation of double-network gel films.

#### 2.2.3. XRD Analysis 

As shown in Figure 1B, the XRD spectra of each gel film were not significantly different, with a broad peak and low intensity between 15 and 35°. Meanwhile, the characteristic peaks of SA and COS disappeared. These results indicate that each gel film was an amorphous material formed by the dominant electrostatic interaction, which was consistent with the FTIR and TGA results [22,23].

#### 2.2.4. Contact Angle

The contact angle measurements of all films were less than 60° (Table 1), reflecting the excellent hydrophilicity of each COS/SA/nM^2+^ PDF, which was suitable for wound recovery [24].

#### 2.2.5. Inflationary Behavior

Good swelling and moisturizing properties are among the most important properties of medical dressings [25]. On the one hand, the excellent swelling property can quickly absorb blood and exudate from the wound to keep the wound clean. On the other hand, the proper swelling ability can keep the wound moist and promote wound healing [26]. 

**Figure 2:**
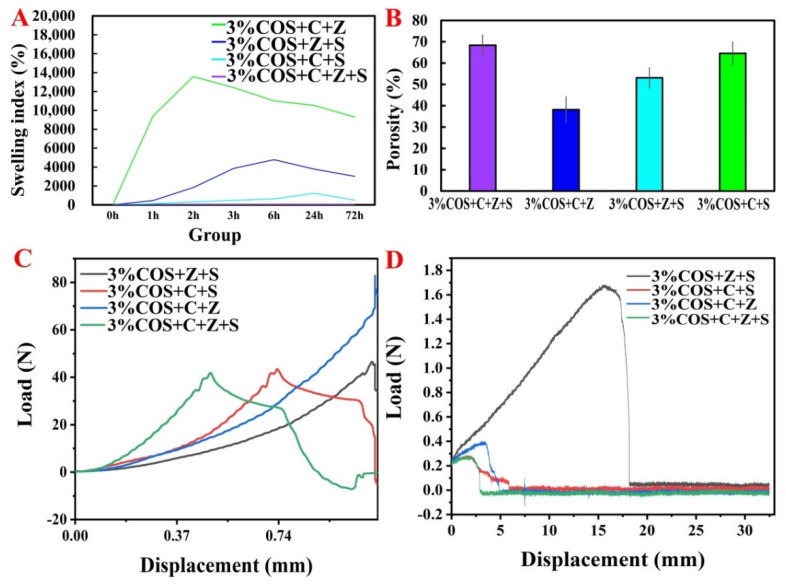
Physical properties of nIon^2+^−COS/SA gel films. (**A**) Dissolution rate of nIon^2+^−COS/SA gel films. (**B**) Porosity of nIon^2+^−COS/SA gel films. (**C**) Tensile strength and deformation curves of nIon^2+^−COS/SA gel films. (**D**) Fracture strength of nIon^2+^−COS/SA gel films.

The swelling rate of all four groups of gel films showed a trend of increasing and then decreasing. The reason for the decrease may be due to the loss of measured mass due to the dissolution and fragmentation of the gel film after reaching saturation and soaking in the solution for too long. The highest swelling rate was reached in the 3%COS+C+Z group (2 h, 14,000%), followed by the 3%COS+Z+S group (6 h, 4000%), and the lowest in the 3%COS+C+Z+S group (Figure 2A). The reason for the large difference in the swelling level of gel films compounded with different metal ions is the different ability of alginate chains to chelate with different metal ions: Sr^2+^ > Ca^2+^ > Zn^2+^, which is related to the radius of metal ions and the coordination mode of SA and metal ions [27]. Consistent with the SEM analysis results (Figure 1E), the stronger the chelating ability of SA with metal ions, the tighter the cross-linked structure formed under the same conditions. Moreover, the water molecules that can be accommodated are correspondingly reduced, while the diffusion of water molecules becomes more difficult, and therefore the swelling rate decreases [28].

#### 2.2.6. Porosity

The porosity of the wound dressing has a significant effect on the rate of wound healing, as it determines the oxygen permeability and nutrient diffusion capacity directly related to cell proliferation and the wound microenvironment [29]. As shown in Figure 2B, the COS/SA/nM^2+^ PDFs all had high a porosity, which enhanced the water retention capacity of gel films and improved the bonding strength inside the gel, which could effectively stabilize the gel structure. Moreover, it may be one of the reasons for the multi-ion enhanced mechanical properties of hydrogels [30]. The porosities of all the gel films were greater than 35%, while the porosities of the 3%COS+C+Z+S group, the 3%COS+Z+S group and the 3%COS+C+S group were slightly higher than that of the 3%COS+C+Z group, indicating a more compact structure; this result was consistent with the SEM observations.

#### 2.2.7. Thermal Stability

DSC and TGA analyses of the polyionic cross-linked gel films were performed by thermal degradation at a heating frequency of 10 °C/min under nitrogen flow (Figure 1C,D). It could be seen that each COS/SA/nM^2+^PDF lost some weight around 100 °C, which was due to the evaporation of water adsorbed on the surface of COS/SA/nM^2+^ PDF and the detachment of water from the crystalline state. In the temperature range of 180 °C to 350 °C, the weight loss of each COS/SA/nM^2+^ PDF was about 35% of the total weight, which was probably due to the electrostatic interaction between the amino group on COS and the carboxyl group on SA. Of course, it could also be due to the loss of chelation between the metal ions and SA and the decomposition of COS [4]. The electrostatic interactions between the three were also demonstrated. In the temperature range of 350 °C to 800 °C, the gel films lost about 30% of the total weight, and the weight loss could be attributed to the degradation of SA starting and the gradual carbonization and oxidative decomposition with increasing temperature. Meanwhile, compared with the gel films compounded with two metal ions, the gel films compounded with three metal ions (the 3%COS+C+Z+S group) had a stronger stability. Not only was the weight loss rate at each weight loss stage lower than in the remaining three groups, but the rate of weight change with time was also slower.

### 2.3. Mechanical Properties of COS/SA/nM^2+^ PDF

The mechanical properties of the multi-ion cross-linked gel films were tested as shown in Figure 2C,D. As can be seen from the figure, the tensile strengths, from largest to smallest, were the 3%COS+C+Z+S group, 3%COS+C+S group, 3%COS+Z+S and 3%COS+C+Z group. The better mechanical properties of each group could be attributed to the addition of metal ions that increased the adhesion of the surface [31]. Moreover, Zn^2+^ and Sr^2+^ can cross-link with M,G units in SA by non-selective coordination to form a uniform and dense gel structure [32]. Each COS/SA/polymetallic ion complex hydrogel film exhibits diverse mechanical properties to meet the different needs of dressing mechanical properties for different wounds.

### 2.4. Antioxidant Activity of COS/SA/nM^2+^ PDF

**Figure 3:**
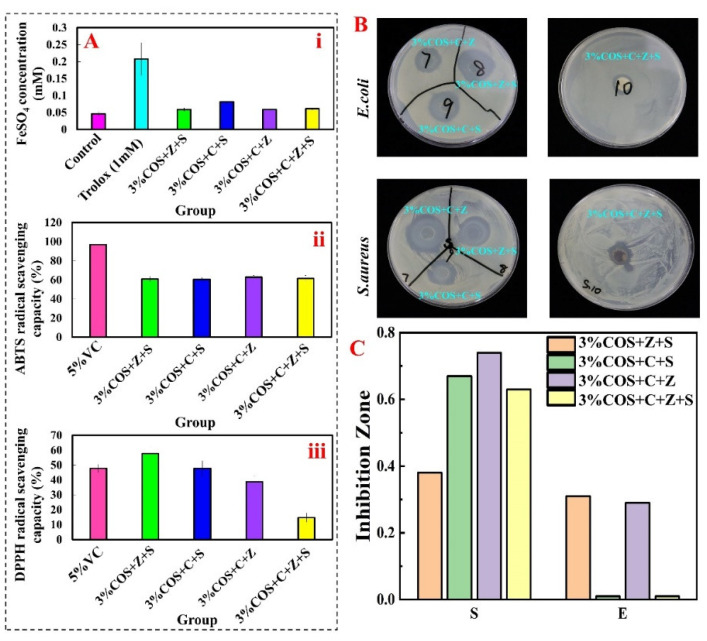
Activity evaluation of nIon^2+^−COS/SA gel films in vitro. (**A**) In vitro antioxidant performance evaluation of nIon^2+^−COS/SA gel films. From top to bottom, they are Fe^2+^ chelating activity (i), DPPH radical scavenging activity (ii), and ABTS radical scavenging activity (iii). (**B**) Physical diagram of the inhibition circle of nIon^2+^−COS/SA gel films. (**C**) Histogram of the inhibition circle of nIon^2+^−COS/SA gel films.

Hydroxyl radicals are the most active free radicals and can react with most biomolecules including proteins, lipids and nucleic acids [33]. Fe^2+^ chelating ability, ABTS radical scavenging activity and DPPH radical scavenging activity were selected to evaluate the antioxidant properties of gel films [34,35]. Analysis of the in vitro antioxidant activity results in Figure 3A revealed that the multi-ionic cross-linked gel films had some scavenging and reducing ability for all three, and the 3%COS+Z+S group had a significant scavenging effect on DPPH radicals, even higher than the positive control Vitamin C, while that of the 3%COS+C+Z+S group was relatively poor. Compared with scavenging DPPH radicals, the scavenging ability of the four groups of gels for ABTS was approximately the same, but there was a gap with the positive control group. The good antioxidant property also proved its potential as a wound dressing.

### 2.5. Antibacterial Properties of COS/SA/nM^2+^ PDF

The antimicrobial properties of ionic cross-linked hydrogels represent an important feature in the prevention of infection preventive wound care without the use of antibiotics. In Figure 3B, numbers 7, 8, 9 and 10 correspond to the 3%COS+C+Z group, 3%COS+Z+S group, 3%COS+C+S group and 3%COS+C+Z+S group in turn. The inhibition circles formed by the 3%COS+Z+S and 3%COS+C+S groups were larger (Diameter in *E. coli*: 2.49 cm, 2.82 cm; in *S. aureus*: 2 cm, 1.93 cm). That is, both of them have stronger inhibition ability for *E. coli* and *S. aureus*. Compared to the 3%COS+C+Z+S group, the inhibition effect of the 3%COS+C+Z group (in *E. coli*: 1.62 cm; in *S. aureus*: 2.9 cm) was significantly better. The stronger antibacterial effect of the multi-ionic cross-linked gel film prepared in this study has potential as a wound dressing field.

### 2.6. Cytocompatibility of COS/SA/nM^2+^ PDF

**Figure 4:**
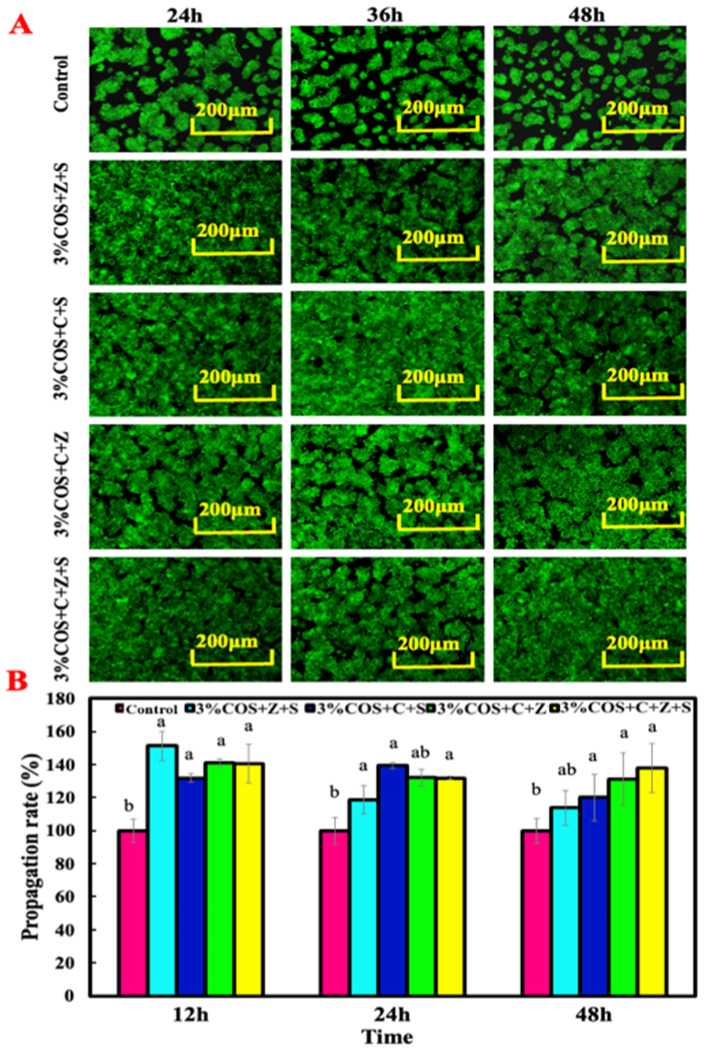
In vitro cytotoxicity test of nIon^2+^–COS/SA gel film extracts. (**A**) HaCaT AO/EB fluorescence staining for 24 h, 36 h and 48 h (magnification: ×5). Cells fluoresce green after acridine orange (AO) crosses the intact cell membrane and embeds in nuclear DNA. Ethidium bromide (EB) penetrates damaged membranes, interacts with DNA and fluoresces orange-red. (**B**) Cell viability was assayed by CCK-8 assay. HaCaT cells were maintained for 12 h, 24 h and 48 h under the conditions described. Note: Same superscript letters indicate no significant difference (*p* > 0.05), different superscript letters indicate significant difference (*p* < 0.05).

For skin wound healing, cell proliferation is important for wound contraction and later healing stages [36,37,38]. It is clear from Figure 4A that the nIon^2+^-COS/SA gel films were all biocompatible. After incubation of HaCaT with gel film extracts for 24 h, 36 h, and 48 h, the cells were stained green and did not show any red-orange color. This means that HaCaT cells grew well in all groups of gel films, verifying their good biocompatibility to enhance cell viability. More interestingly, it was further demonstrated on the results of electron microscopy tests (Figure 1E) that the higher specific surface area and water content can provide some three-dimensional space for cell adhesion and proliferation. 

In CCK-8 experiments, since dead or damaged cells do not contain dehydrogenase activity, the products generated are proportional to the number of cells and can be used to estimate the number of living cells present (Figure 4B). The four groups of gel films slowed down the proliferation of HaCaT cells at 12 h, 24 h and 48 h, with an overall decreasing trend, but all were higher than the control group. At 12 h, all four groups of samples significantly promoted cell proliferation (*p* < 0.05). At 24 h, the 3%COS+C+S group significantly promoted cell proliferation compared with the other groups (*p* < 0.05). At 48 h, the 3%COS+C+Z+S group had the best efficacy (*p* < 0.05). Overall it was consistent with the results of the AO/EB double staining assay, which further verified its good cytocompatibility.

### 2.7. In Vivo Healing Promotion Experiment of COS/SA/nM^2+^ PDF

Essentially, the porosity of the dressing determines oxygen penetration and nutrient diffusion directly related to the wound microenvironment and cell proliferation, which can affect the rate of wound healing. Before performing in vivo wound healing, the porosity of COS/SA/nM^2+^ PDF was measured (Figure 2B), and the 3%COS+Z+S group was found to have a higher porosity and other physicochemical properties that were relatively superior. Therefore we selected the 3%COS+Z+S group as the dressing material for in vivo healing promotion experiments.

#### 2.7.1. Wound Healing Assessment

**Figure 5:**
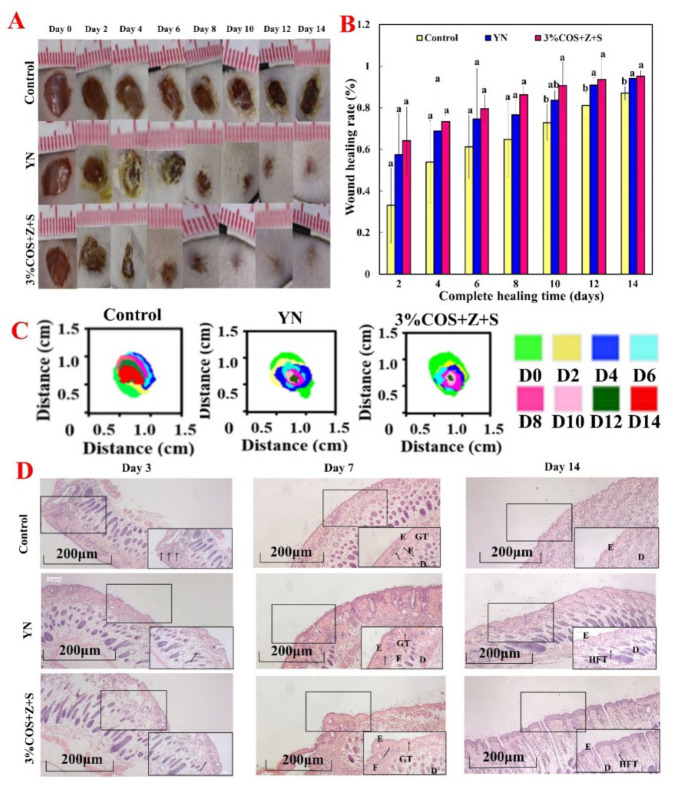
Evaluation of wound healing ability of nIon^2+^−COS/SA gel films. NIon^2+^−COS/SA gel film is shown in A−C for the evaluation of wound defects in mice. (**A**) Representative photographs of wounds in mice on days 0, 2, 4, 6, 8, 10, 12 and 14. (**B**) Wound healing rate of each group (calculated every two days). Values are expressed as mean ± standard deviation, n = 3. Note: Same superscript letters indicate non-significant differences (*p* > 0.05), different superscript letters indicate significant differences (*p* < 0.05). (**C**) Simulated changes in wound size and morphology at 8 time points. (**D**) Representative images of H&E staining (5×). Note: Thick black arrows indicate inflammatory cell infiltration. Letters D, E, F, GT and HFT represent dermis, epidermis, fibroblasts, granulation tissue and hair follicle tissue, respectively.

The ability of different ionic cross-linked hydrogels to heal total skin defects was evaluated in a total cortical wound model based on the results of in vitro studies. Skin wound repair is divided into four phases: inflammation, granulation tissue formation, tissue remodeling and re-epithelialization [39]. In vivo wound healing ability by observing the macroscopic wound closure within 14 days was verified [40] (Figure 5). The macroscopic appearance photos of the wounds at different times after injury are shown in Figure 5A. The gel films of the 3%COS+Z+S group could accelerate the wound repair in the mouse model. After 3 days of treatment, the gel films of the 3%COS+Z+S group showed a decrease in inflammatory cells compared with the control group, indicating an earlier wound healing process, a decrease in the inflammatory process as well as in the number of inflammatory cells. And this was closely related to the significant antibacterial ability and anti-inflammatory properties of Zn^2+^ [41]. On day 8 and beyond, granulation tissue formation was observed on the wounds of COS/SA/nM^2+^ PDF, and the percentage of wound closure was significantly reduced (at day 8: 86.24%), with essentially complete healing on day 14. The 3%COS+Z+S group was significantly different from the blank group on both day 12 and day 14 (*p* < 0.05), but was not significantly different from the positive control group (*p* > 0.05), which also further demonstrated the good biocompatibility shown by the cellular assay.

#### 2.7.2. Histological Evaluation of Regenerated Tissue

The histological results confirmed the observation of optical images of wounds (Figure 5D). H&E staining showed that the 3%COS+Z+S group gel film had formed a neo-epidermal layer on day 7, with a significant increase in neovascularization in the regenerated tissue of the treated wounds. Zinc and strontium ions were reported to promote the release of angiogenic factors such as vascular endothelial growth factor, basic fibroblast growth factor and matrix metalloproteinase-2 in tissue-engineered bone [42]. It has also been reported that strontium released from biomaterials enhances the proliferation and migration of endothelial cells and promotes the formation of tubular structures in vitro [43]. This is consistent with the results in Figure 4A.

#### 2.7.3. Expression of FGF and CD31 during Wound Healing

The above experiments initially determined the wound healing-promoting ability of the gel films in the 3%COS+Z+S group, and this conclusion was further confirmed by immunohistochemical analysis. Compared with the other two groups, the gel films of 3%COS+Z+S group showed the highest content (16.82%) at day 7 and lower content at day 14, which proved that fibroblast growth factor was more in the cell proliferation phase and less in the tissue remodeling phase [44], consistent with the results of cellular experiments.

**Figure 6:**
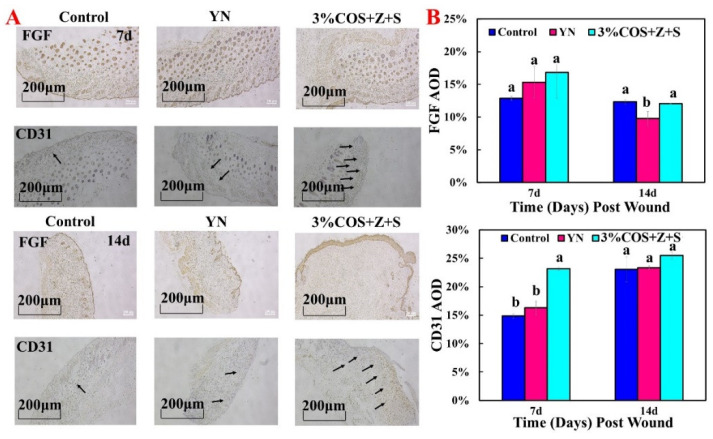
Expression of FGF and CD31 of nIon^2+^−COS/SA gel films in mice. (**A**) From top to bottom, representative images of FGF and CD31 analysis on day 7; representative images of FGF and CD31 analysis on day 14. The black arrow indicated the expression of CD31. (**B**) Quantitative immunohistochemical analysis on day 7 and day 14. Note: Same superscript letters indicate non−significant differences (*p* > 0.05), different superscript letters indicate significant differences (*p* < 0.05).

CD31 is a marker of vascular endothelial cells and appears as yellow or brownish yellow in immunohistochemistry. As shown in Figure 6, the AOD values of CD31 were highest on day 7 and day 14 compared with the other two groups (7d:23.18%; 14d:25.48%) and were significantly different from the other two groups at day 7 (*p* < 0.05). In a nutshell, it demonstrates the potential of the hydrogel film of the 3%COS+Z+S group to promote wound healing rapidly in a short period of time.

## 3. Materials and Methods

### 3.1. Materials

Sodium alginate (SA, M_w_ = 460,000 g · mol^−1^), chitooligosaccharide (COS, molecular weight < 2000 Da), SrCl_2_, CaCl_2_, and ZnCl_2_ were purchased from Shanghai Maclean Biochemical Co. (Shanghai, China). Yunnan Baiyao (YN) was purchased from Yunnan Baiyao Group Co. (Kunming, China). The acridine orange (AO)/ethidium bromide (EB) staining kit was purchased from Biotechnology (Shanghai, China) Co. Cell Counting Kit-8 (CCK-8) was purchased from APExBIO (Houston, TX, USA). H&E staining kits was purchased from Solaibao (Beijing, China). *Escherichia coli* (*E. coli*, CCTCCAB93154) and, *Staphylococcus aureus* (*S. aureus*, ATCC9118) were purchased from China Type Culture Collection Center. HaCaT cells were purchased from Suzhou Benetton Biotechnology Co. Ltd. (Suzhou, China). SPF weight 20 ± 2 g male mice were purchased from Hebei Fu (Beijing, China) Biotechnology Co., Ltd. (Beijing, China) 20190010. All chemicals were analytical degree reagents and used as received. 

### 3.2. Preparation Process of nIon^2+^-COS/SA Gel Films

The specific preparation process of nIon^2+^-COS/SA gel films is as follows.

(1) A certain amount of COS and SA was weighed and deionized water was added to prepare a mass fraction of 3% COS with a mass fraction of 2% SA.
(2) The SA solution with a mass fraction of 2% was heated at 90°C for 3 h. The COS solution was adjusted to pH 5.6 with GDL and then stirred at room temperature for 1 h.
(3) CaCl_2_-ZnCl_2_, CaCl_2_-SrCl_2_, SrCl_2_-ZnCl_2_, and CaCl_2_-ZnCl_2_-SrCl_2_ powders with each metal ion concentration of 1% were added to the 3% COS solution and stirred at room temperature for 5 min.
(4) After that, 12 mL of SA solution with 2% mass fraction was poured into the prefabricated mold (The length, width and height were 10 cm, 5 cm and 1 cm respectively, for the Teflon plane) by the extended flow method.
(5) The solution in step (4) was loaded into a medical spray bottle, left to remove air bubbles, and then sprayed evenly on the surface of the SA solution at a distance of 15–20 cm from the mold. The thickness of the composite hydrogel can be controlled by the sprayed solution due to the height limitation of the mold with the adjustment of the number of sprays. After extensive testing, it was determined that the number of coats required to fully cross-link the hydrogel and to form a more uniform film was 15.
(6) After spraying, the molds were left to stand for 10 min, and the COS/SA/nM^2+^ PDFs were gradually formed. These gel films were named as 3%COS+C+Z, 3%COS+C+S, 3%COS+Z+S and 3%COS+C+Z+S, respectively.

### 3.3. SEM Morphological Analysis

After gold spraying of suitably sized lyophilized gel films, a SEM (Nova NanoSEM 450, FEI, Hillsboro, OR, USA) field emission scanning electron microscope (FESEM) equipped with an energy dispersive X-ray spectrometer (X-MaxN50, OXFORD) was used to observe the microstructure and morphology, as well as to collect energy dispersive spectra (EDS) (Quanta 250 FEG, FEI, Hillsboro, OR, USA).

### 3.4. Fourier Transform Infrared Spectroscopy (FTIR) 

The infrared absorption spectra of each COS/SA/nM^2+^ PDF were measured using an IRSpirit-1 instrument (FTIR, Shimadzu, Kyoto, Japan) at a resolution of 4 cm^−1^ and a scanning frequency of 32 times.

### 3.5. XRD

The X-ray photoelectron spectra of each COS/SA/nM^2+^ PDF were measured by an X-ray diffractometer (Ultima, Rigaku, Tokyo, Japan) at an angular resolution of 0.02° and a diffraction angular velocity of 10°/min in the 2θ range of 20° to 80°.

### 3.6. Contact Angle

To examine the hydrophilicity of the films, the static contact angle of each COS/SA/nM^2+^ PDF was measured by a contact angle meter (OCA20, Dataphysics, Filderstadt, Germany) immediately after droplet deposition.

### 3.7. Inflationary Behavior

The mass of the lyophilized films was weighed and recorded as *W*0. The lyophilized films were incubated in deionized water at room temperature, removed at regular intervals, and weighed immediately after wiping off the excess water adhering to the surface with filter paper, and the mass was recorded as *Wt*. The process was repeated until the gel films reached a state of swelling equilibrium and the mass did not change. All experiments were repeated three times for each group.

The solubility (*Q*) of the hydrogel film is calculated as follows.

$$Q\%=\frac{Wt-W0}{W0}\times 100\%$$

### 3.8. Porosity Measurements

The porosity of the films was measured using the liquid displacement method. Each hydrogel film was cut into a fixed shape of 1 cm × 1 cm, and the weight of the hydrogel film was weighed. The gel films were placed in a beaker containing 5 mL of anhydrous ethanol for 24 h until the swelling equilibrium was reached, and then the gel films were removed, the filter paper was wiped to remove excess alcohol, and they were weighed. The porosity of COS/SA/nM^2+^ PDF was calculated as follows.

$$Porosity\%=\frac{W2-W1-W3}{W2-W3}\times 100\%$$

where *W*1 is the initial weight of the hydrogel film, *W*2 is the weight of the hydrogel film in the dissolved equilibrium state, and *W*3 is the weight of anhydrous ethanol after removing the hydrogel film.

### 3.9. Thermal Stability

The thermogravimetric curves of each COS/SA/nM^2+^ PDF were measured by heating the lyophilized gel films from 50 °C to 800 °C at a heating rate of 10 °C/min using a simultaneous thermal analyzer (STA 449C, TA Instruments, DuPont, New Castle, DE, USA) under nitrogen protection at a flow rate of 50 mL/min.

### 3.10. Mechanical Properties

The polymer films were cut into rectangular specimens of 20 mm × 10 mm, and each COS/SA/nM^2+^ PDF was tested in tension using a mass spectrometer (TMS-PRO, FTC, West Sussex, VA, USA) at a stretching rate of 2 mm/min.

### 3.11. Antioxidant Activity Analysis

#### 3.11.1. FRAP Measurement

An aliquot of 5 μL of each COS/SA/nM^2+^ PDF and 180 μL of FRAP reagent were mixed in a 96-well plate and incubated at 37 °C for 5 min before measuring the absorbance of the mixture at 593 nm using an enzyme marker (VersaMax, Molecular Devices, Silicon Valley, CA, USA). Vitamin E was used as a positive control.

#### 3.11.2. DPPH Free Radical Scavenging Activity

A total of 2 mL of the extract of each COS/SA/nM^2+^ PDF was mixed with 2 mL of DPPH solution (0.1 mM, 95% methanol) and incubated for 30 min at room temperature in the dark. Afterwards, the absorbance of the mixture at 517 nm was measured using an enzyme marker (VersaMax, Molecular Devices, Silicon Valley, CA, USA). Vitamin C was used as a positive control.

#### 3.11.3. ABTS Free Radical Scavenging Ability

ABTS^+^ solution was prepared by mixing 0.7 mM of ABTS solution with an equal volume of 2.45 mM of potassium persulfate solution. The ABTS^+^ solution was incubated for 16 h at room temperature and protected from light, and then diluted with phosphate buffer solution (5.0 mM, pH 7.4) to obtain an absorbance of 0.70 ± 0.05 at 734 nm. Subsequently, a 5.0 μL aliquot of the diluted ABTS^+^ solution was added to 0.2 mL of each COS/SA/nM^2+^ PDF in the extraction solution. After incubation for 10 min, the absorbance of the mixture was measured at 734 nm using an enzyme marker (VersaMax, Molecular Devices, Silicon Valley, CA, USA). Phosphate buffer solution was used as a control.

### 3.12. Antibacterial Activity Analysis

The antibacterial performance of each nIon^2+^-COS/SA hydrogel films against Gram-negative and Gram-positive bacteria was determined by the inhibition circle method [45]. The strains used for the experiments were *E. coli* and *S. aureus*. A total of add 100 μL of Escherichia coli and Staphylococcus aureus solutions preserved in glycerol were added, respectively, to 10 mL of LB liquid medium (NaCl 10.0; yeast extract 5.0; tryptone 10.0 g·L^−1^). After 10 h of shaking incubation in an oscillating incubator at 37 °C and 100 rpm, the optical density was measured with a spectrophotometer at 600 nm wavelength to assess bacterial growth. After several centrifugations and rinses, E. coil bacterial suspension and S. aureus bacterial suspension were respectively resuspended to 1.0 × 10^8^ CFU/mL with PBS buffer. Bacterial suspensions of *E. coli* and *S. aureus* (100 μL) were evenly spread on agar solid plates using an L-shaped applicator, after which a uniformly sized hydrogel of 1.5 cm in diameter was placed in the center. After 1 d of incubation under conventional conditions, the diameter of the circular transparent area around the hydrogel was measured.

### 3.13. Cellular Experiments

#### 3.13.1. Cell Viability Assay

Gel films of 1 cm × 1 cm were first incubated overnight in complete medium and filtered through a film, and then HaCaT cells were inoculated into 96-well plates containing this complete medium at a density of 10,000 cells/well. The 96-well plates were incubated in an incubator at 37 °C with 5% CO_2_. AO/EB mixed fluorescent staining solution was prepared by mixing equal amounts of equal concentrations of AO solution and EB solution. After the HaCaT cells were, respectively, cultured to 24 h, 36 h, and 48 h, the HaCaT cells were dissociated by adding appropriate amount of trypsin in the 96-well plate. Then, 20 μL of HaCaT cell suspension was aspirated and an appropriate amount of AO/EB mixed fluorescent staining solution was added, and the morphology of HaCaT cells on the slide was observed using a fluorescent inverted microscope.

#### 3.13.2. Cell Proliferation Assay

HaCaT cells were cultured in DMEM/F12 medium containing 10% fetal bovine serum (FBS) at 37 °C. Briefly, HaCaT cells were added to 96-well plates at a density of 2 × 10^5^ cells/well and incubated for 4 h for adhesion. Then, 10 μL of filtered hydrogel film extract was added to each well, and the original medium was replaced with 200 μL of serum-free DMEM cell medium containing 10% CCK-8 reagent at 12 h, 24 h, and 48 h again. After a total of 90 min incubation in an incubator, the liquid was transferred to a 96-well plate and the optical density at 450 nm was measured with an enzyme marker.

### 3.14. In Vivo Healing Promotion Experiment

Based on the above performance characterization, the gel film with the best overall performance was selected for the next step of in vivo healing promotion experiments.

#### 3.14.1. Laboratory Animals

All animal experiments in this paper followed the regulations of the Animal Experiment Center of Shandong University. The 27 mice (SPF-grade male ICR mice with a mass of 20 ± 5 g) used in the experiments were divided into 3 groups, including the blank group, positive control (Yunnan Baiyao) group, and 3%COS+Z+S group. All mice were acclimatized and fed for three days before conducting the experiments to familiarize them with their environment.

#### 3.14.2. Trauma Modeling

The mice were anesthetized intraperitoneally with a 1% pentobarbital solution at a dosage of 5 mL/kg, and the hair on their backs was removed with a shaver and some of the remaining fine hair was removed with a 10% mass fraction of sodium sulfide solution. Then, 75% alcohol was used to disinfect the back of the mice and a full skin wound of 0.8 cm in diameter was created with a punch. The UV-sterilized hydrogel film dressing was sutured to the full skin wound on the back of the mice and all mice were separately fed in a single cage.

#### 3.14.3. Animal observation

Wound status was recorded every two days from the date of establishment of the mouse trauma model using a digital camera to take photographs. Wound healing rates were calculated using Image J (National Institutes of Health, Bethesda, MD, USA) with the following equation.

$$Wound\;healing\;rate\%=\frac{D0-Df}{D0}\times 100\%$$

where *D*0 is the diameter of the full dorsal wound of the mouse at the time of establishment, and *Df* is the final diameter of the dorsal wound of the mouse at the experimental stage.

#### 3.14.4. Histological Assessment

Regenerated skin tissue specimens were respectively collected on days 3, 7 and 14 after injury. Tissue sections were fixed with 4% paraformaldehyde and embedded with paraffin. The sections were stained with H&E and then observed with a microscope (Axio Observer, Zeiss, Baden-Württemberg, Germany).

#### 3.14.5. Immunohistochemical Analysis

The expression of fibroblast growth factor-10 (FGF-10) and platelet-endothelial cell adhesion molecules (CD31) was detected by immunohistochemistry. Tissue sections were collected, dewaxed, and placed in distilled water to repair antigens. Endogenous peroxidase was blocked by 3% H_2_O_2_-methanol solution, and antigen was recovered with citrate buffer (pH 6.0). Sections were sealed and incubated with 50–100 μL primary antibody (diluted 50–100 μL primary antibody) for 2 h at 37 °C in a humid atmosphere. They were washed with PBS and incubated at 37 °C for 30 min, and then a universal IgG Antibody-Fab fragment-HRP multimer (50 μL) was added. Sections were viewed under a light microscope (Axio Observer, Zeiss, Baden-Württemberg, Germany) and the photographs were later analyzed using Image J (National Institutes of Health, Bethesda, Staten Island, New York, NY, USA).

### 3.15. Statistical Analysis

The experimental data were expressed as mean ± standard deviation (Average ± S.D). SPSS (version 20) was used for statistical analysis. Multiple comparisons were performed between groups using the LSD method with a significance level of *p* < 0.05. Wound area was calculated using Image J (National Institutes of Health, Bethesda, Staten Island, New York, NY, USA) based on experimental images.

## 4. Conclusions

In this study, we rapidly synthesized and characterized sprayable nIon^2+^-COS/SA gel films by the casting and in -situ spray method. The mechanical properties and biocompatibility were improved by introducing divalent ions into the hydrogels to synthesize the advantages of these metal ions in a complex manner. Among the four hydrogels, zinc cross-linked hydrogels showed significant antibacterial ability, cell viability against *S. aureus* and *E. coli*, and the best overall performance in the 3%COS+Z+S group. Thus, we performed in vivo healing promotion experiments to further verify their efficacy. It was demonstrated that the gel films of the 3%COS+Z+S group had a superior ability to increase the percentage of wound closure, collagen deposition, granulation tissue and angiogenesis. In conclusion, the COS/SA/nM^2+^ PDF is not only easy to prepare and naturally harmless, but also has good antimicrobial and biocompatible properties, which brings new insights into the design of safe, efficient, and multifunctional wound dressings that can be prepared by rapid spraying.

## Figures and Tables

**Table 1:** Contact angle of nIon^2+^-COS/SA gel films.

Group	Contact Angle
3%COS+C+S	33.1°
3%COS+C+Z	55.2°
3%COS+Z+S	46.1°
3%COS+C+Z+S	38.2°

## Data Availability

Data is contained within the article.

## References

[1] Coles M.C., Buckley C.D. (2019). Ready-made cellular plugs heal skin wounds. Nature.

[2] Parfejevs V., Debbache J., Shakhova O., Schaefer S.M., Glausch M., Wegner M., Suter U., Riekstina U., Werner S., Sommer L. (2018). Injury-activated glial cells promote wound healing of the adult skin in mice. Nat. Commun..

[3] An Y., Lin S.Y., Tan X.J., Zhu S.O., Nie F.F., Zhen Y.H., Gu L.S., Zhang C.L., Wang B.C., Wei W. (2021). Exosomes from adipose-derived stem cells and application to skin wound healing. Cell Proliferat.

[4] Ailincai D., Rosca I., Morariu S., Mititelu-Tartau L., Marin L. (2022). Iminoboronate-chitooligosaccharides hydrogels with strong antimicrobial activity for biomedical applications. Carbohydr. Polym..

[5] Chandika P., Kim M.S., Khan F., Kim Y.M., Heo S.Y., Oh G.W., Kim N.G., Jung W.K. (2021). Wound healing properties of triple cross-linked poly (vinyl alcohol)/methacrylate kappa-carrageenan/chitooligosaccharide hydrogel. Carbohydr. Polym..

[6] Chen Y., Ling Z., Mamtimin T., Khan A., Peng L., Yang J., Ali G., Zhou T., Zhang Q., Zhang J. (2022). Chitooligosaccharides production from shrimp chaff in chitosanase cell surface display system. Carbohydr. Polym..

[7] Wang S., Luo Y., Huang L., Wang S., Hao C., Sun L., Zhang Y., Wang W., Li C. (2022). The inhibition effects and mechanisms of sulfated chitooligosaccharides on influenza A virus in vitro and in vivo. Carbohydr. Polym..

[8] Ohm Y., Pan C.F., Ford M.J., Huang X.N., Liao J.H., Majidi C. (2021). An electrically conductive silver-polyacrylamide-alginate hydrogel composite for soft electronics. Nat. Electron..

[9] Suzuka J., Tsuda M., Wang L., Kohsaka S., Kishida K., Semba S., Sugino H., Aburatani S., Frauenlob M., Kurokawa T. (2021). Rapid reprogramming of tumour cells into cancer stem cells on double-network hydrogels. Nat. Biomed. Eng..

[10] Matsumae G., Terkawi M.A., Nonoyama T., Kurokawa T., Takahashi D., Shimizu T., Kadoya K., Gong J.P., Yasuda K., Iwasaki N. (2022). Evaluation of biological responses to micro-particles derived from a double network hydrogel. Biomater. Sci..

[11] Wu S., Lou D.Y., Wang H.Y., Jiang D.Q., Fang X., Meng J.Q., Sun X.Y., Li J. (2022). One-pot synthesis of anti-freezing carrageenan/polyacrylamide double-network hydrogel electrolyte for low-temperature flexible supercapacitors (vol 435, 135057, 2022). Chem. Eng. J..

[12] Ramdhan T., Ching S.H., Prakash S., Bhandari B. (2022). Evaluation of alginate-biopolymers (protein, hydrocolloid, starch) composite microgels prepared by the spray aerosol technique as a carrier for green tea polyphenols. Food Chem..

[13] Zhou Z.B., Xiao J.W., Guan S.W., Geng Z.J., Zhao R.F., Gao B.T. (2022). A hydrogen-bonded antibacterial curdlan-tannic acid hydrogel with an antioxidant and hemostatic function for wound healing. Carbohydr. Polym..

[14] Meng L., Ma J., Liu C., Mao X., Li J. (2022). The microbial stress responses of Escherichia coli and Staphylococcus aureus induced by chitooligosaccharide. Carbohydr. Polym..

[15] Yue H.Y., Shang Z.J., Xu P., Feng D.Y., Li X.X. (2022). Preparation of EDTA modified chitooligosaccharide/sodium alginate/Ca2+ physical double network hydrogel by using of high-salinity oilfield produced water for adsorption of Zn2+, Ni2+ and Mn2+. Sep. Purif. Technol..

[16] Zhang M., Qiao X.N., Han W.W., Jiang T.Z., Liu F., Zhao X. (2021). Alginate-chitosan oligosaccharide-ZnO composite hydrogel for accelerating wound healing. Carbohydr. Polym..

[17] Lee H., Seo Y.J., Kim J., Bae M.J., Hwang S., Bae J.G., Lee W.B., Yoon H. (2022). Function transformation of polymeric films through morphing of surface shapes. Chem. Eng. J..

[18] Griveau L., Lafont M., le Goff H., Drouglazet C., Robbiani B., Berthier A., Sigaudo-Roussel D., Latif N., Visage C.L., Gache V. (2022). Design and characterization of an in vivo injectable hydrogel with effervescently generated porosity for regenerative medicine applications. Acta Biomater..

[19] Wang X.Y., Kim H.J. (2022). Ultra-stretchable dual-network ionic hydrogel strain sensor with moistening and anti-freezing ability. Prog. Org. Coat..

[20] Rettke D., Danneberg C., Neuendorf T.A., Kuhn S., Friedrichs J., Hauck N., Werner C., Thiele J., Pompe T. (2022). Microfluidics-assisted synthesis and functionalization of monodisperse colloidal hydrogel particles for optomechanical biosensors. J. Mater. Chem. B.

[21] Kamal T., Ul-Islam M., Khan S.B., Bakhsh E.M., Chani M.T.S. (2022). Preparation, Characterization, and Biological Features of Cactus Coated Bacterial Cellulose Hydrogels. Gels.

[22] Lakouraj M.M., Mojerlou F., Zare E.N. (2014). Nanogel and superparamagnetic nanocomposite based on sodium alginate for sorption of heavy metal ions. Carbohydr. Polym..

[23] Hoang H.T., Vu T.T., Karthika V., Jo S.H., Jo Y.J., Seo J.W., Oh C.W., Park S.H., Lim K.T. (2022). Dual cross-linked chitosan/alginate hydrogels prepared by Nb-Tz ‘click’ reaction for pH responsive drug delivery. Carbohydr. Polym..

[24] Mondal M.I.H., Haque M.O., Ahmed F., Pervez M.N., Naddeo V., Ahmed M.B. (2022). Super-Adsorptive Biodegradable Hydrogel from Simply Treated Sugarcane Bagasse. Gels.

[25] Shen G.C., Gao K.P., Zhao N., Yi Z.R., Jiang C.P., Yang B., Liu J.Q. (2021). A novel flexible hydrogel electrode with a strong moisturizing ability for long-term EEG recording. J. Neural Eng..

[26] Kapanya A., Somsunan R., Phasayavan W., Molloy R., Jiranusornkul S. (2021). Effect of molecular weight of poly(ethylene glycol) as humectant in interpenetrating polymer network hydrogels based on poly(sodium AMPS) and gelatin for wound dressing applications. Int. J. Polym. Mater. Polym. Biomater..

[27] Pal V.K., Roy S. (2022). Cooperative Metal Ion Coordination to the Short Self-Assembling Peptide Promotes Hydrogelation and Cellular Proliferation. Macromol. Biosci..

[28] Cao L.Q., Lu W., Mata A., Nishinari K., Fang Y.P. (2020). Egg-box model-based gelation of alginate and pectin: A review. Carbohydr. Polym..

[29] Siboro S.A.P., Anugrah D.S.B., Ramesh K., Park S.H., Kim H.R., Lim K.T. (2021). Tunable porosity of covalently crosslinked alginate-based hydrogels and its significance in drug release behavior. Carbohydr. Polym..

[30] Lin Z.K., Yang Y.R., Liang Z.Z., Zeng L., Zhang A.P. (2021). Preparation of Chitosan/Calcium Alginate/Bentonite Composite Hydrogel and Its Heavy Metal Ions Adsorption Properties. Polymers.

[31] Shaheen A., Maswal M., Dar A.A. (2021). Synergistic effect of various metal ions on the mechanical, thixotropic, self-healing, swelling and water retention properties of bimetallic hydrogels of alginate. Colloids Surf. A-Physicochem. Eng. Asp..

[32] Wang C.C., Yang Y.L. (2022). Tunable volume memory poly(acrylic acid sodium) hydrogel by metal ionsd. Funct. Mater. Lett..

[33] Shang K., Tao L.X., Jiang S.Y., Yan J.H., Hu S.K., Yang G.W., Ma C., Cheng S., Wang X.F., Yin J. (2022). Highly flexible hydrogel dressing with efficient antibacterial, antioxidative, and wound healing performances. Biomater. Sci..

[34] Ramirez-Garcia O., Salinas-Moreno Y., Santillan-Fernandez A., Sumaya-Martinez M.T. (2021). Screening antioxidant capacity of Mexican maize (*Zea mays* L.) landraces with colored grain using ABTS, DPPH and FRAP methods. Cereal Res. Commun..

[35] Jo Y.J., Cho H.S., Chun J.Y. (2021). Antioxidant activity of beta-cyclodextrin inclusion complexes containing trans-cinnamaldehyde by DPPH, ABTS and FRAP. Food. Sci. Biotechnol..

[36] Kim S., Ko J., Choi J.H., Kang J.Y., Lim C., Shin M., Lee D.W., Kim J.W. (2022). Antigen-Antibody Interaction-Derived Bioadhesion of Bacterial Cellulose Nanofibers to Promote Topical Wound Healing. Adv. Funct. Mater..

[37] Pei M.J., Peng X.T., Wan T.T., Fan P.H., Yang H.J., Liu X., Xu W.L., Zhou Y.S., Xiao P. (2021). Double cross-linked poly(vinyl alcohol) microcomposite hydrogels with high strength and cell compatibility. Eur. Polym. J..

[38] Liu S., Zhang Z.M., Zhang J.Q., Qin G.W., Zhang E.L. (2021). Construction of a TiO2/Cu2O multifunctional coating on Ti-Cu alloy and its influence on the cell compatibility and antibacterial properties. Surf. Coat. Technol..

[39] Tang Y.Q., Cai X.Q., Xiang Y.Y., Zhao Y., Zhang X.G., Wu Z.M. (2017). Cross-linked antifouling polysaccharide hydrogel coating as extracellular matrix mimics for wound healing. J. Mater. Chem. B.

[40] Zou C.Y., Lei X.X., Hu J.J., Jiang Y.L., Li Q.J., Song Y.T., Zhang Q.Y., Li-Ling J., Xie H.Q. (2022). Multi-crosslinking hydrogels with robust bio-adhesion and pro-coagulant activity for first-aid hemostasis and infected wound healing. Bioact. Mater..

[41] Banza M., Rutto H. (2022). Continuous fixed-bed column study and adsorption modeling removal of Ni2+, Cu2+, Zn2+ and Cd2+ ions from synthetic acid mine drainage by nanocomposite cellulose hydrogel. J. Environ. Sci. Health Part A Toxic Hazard. Subst. Environ. Eng..

[42] Rajalekshmy G.P., Rekha M.R. (2021). Strontium ion cross-linked alginate-g-poly (PEGMA) xerogels for wound healing applications: In vitro studies. Carbohydr. Polym..

[43] Nardone V., Zonefrati R., Mavilia C., Romagnoli C., Ciuffi S., Fabbri S., Palmini G., Galli G., Tanini A., Brandi M.L. (2015). In Vitro Effects of Strontium on Proliferation and Osteoinduction of Human Preadipocytes. Stem Cells Int..

[44] Sankari L., Fernandes B.L., Rebelatto C.L.K., Brofman P.R.S. (2020). Evaluation of PVA hydrogel as an extracellular matrix for in vitro study of fibroblast proliferation. Int. J. Polym. Mater. Polym. Biomater..

[45] Yan Q., Liu L.L., Wang T., Wang H.N. (2019). A pH-responsive hydrogel system based on cellulose and dopamine with controlled hydrophobic drug delivery ability and long-term bacteriostatic property. Colloid Polym. Sci..

