# Correction: Zhao et al. Multifunctional Gel Films of Marine Polysaccharides Cross-Linked with Poly-Metal Ions for Wound Healing. *Pharmaceuticals* 2022, *15*, 750

**DOI:** 10.3390/ph19060804

**Published:** 2026-05-22

**Authors:** Di Zhao, Chao Shi, Tingting Guo, Kun Zhang, Shenghao Cui, Liqi Chen, Faming Yang, Jingdi Chen

**Affiliations:** Marine College, Shandong University, Weihai 264209, China; mjqyhhdzdys@163.com (D.Z.); shichao19834031211@163.com (C.S.); gtt1314xf@163.com (T.G.); sdtzzk2021@163.com (K.Z.); csh1473@163.com (S.C.); clychee0307@163.com (L.C.)

**Error in Figure**

In the original publication [1], there was a mistake in Figure 4A as published. Upon re-examination of the original data, the authors identified an inadvertent duplication in the assembly of the AO/EB fluorescence images. The experiment has been repeated following the original protocol, and the figure has been updated with representative images from the replicated experiment. The corrected Figure 4A appears below. The authors state that the scientific conclusions are unaffected. This correction was approved by the Academic Editor. The original publication has also been updated.

## Figures and Tables

**Figure 4:**
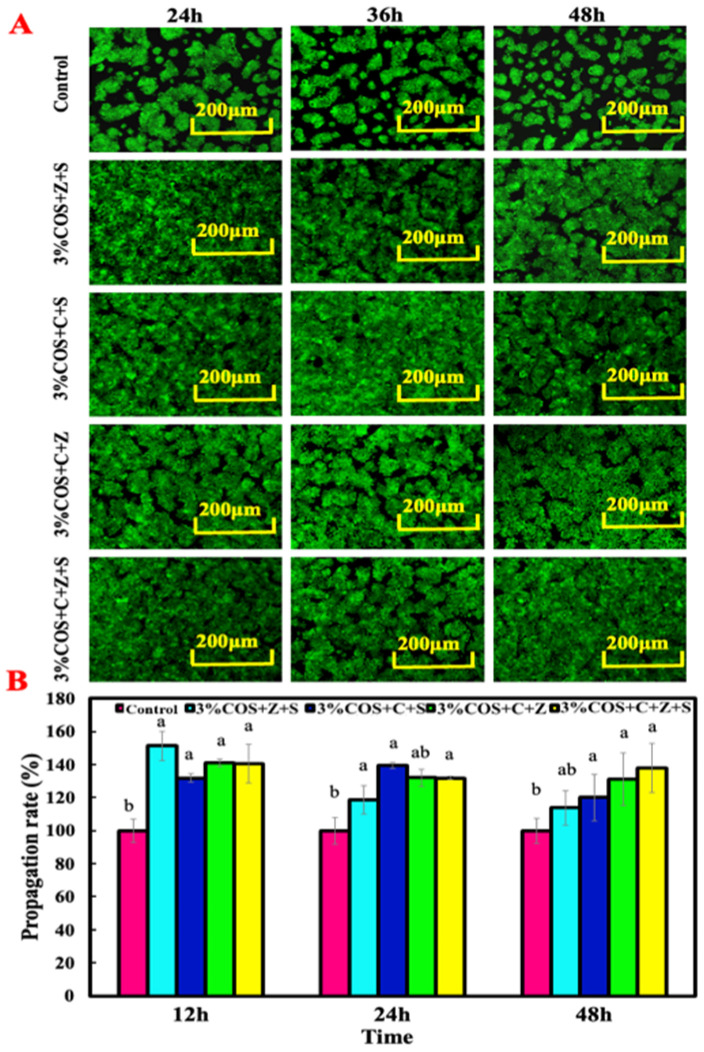
In vitro cytotoxicity test of nIon^2+^–COS/SA gel film extracts. (**A**) HaCaT AO/EB fluorescence staining for 24 h, 36 h and 48 h (magnification: ×5). Cells fluoresce green after acridine orange (AO) crosses the intact cell membrane and embeds in nuclear DNA. Ethidium bromide (EB) penetrates damaged membranes, interacts with DNA and fluoresces orange-red. (**B**) Cell viability was assayed by CCK-8 assay. HaCaT cells were maintained for 12 h, 24 h and 48 h under the conditions described. Note: Same superscript letters indicate no significant difference (*p* > 0.05), different superscript letters indicate significant difference (*p* < 0.05).
